# VHH212 nanobody targeting the hypoxia-inducible factor 1α suppresses angiogenesis and potentiates gemcitabine therapy in pancreatic cancer *in vivo*

**DOI:** 10.20892/j.issn.2095-3941.2020.0568

**Published:** 2021-08-15

**Authors:** Guangbo Kang, Min Hu, He Ren, Jiewen Wang, Xin Cheng, Ruowei Li, Bo Yuan, Yasmine Balan, Zixuan Bai, He Huang

**Affiliations:** 1 Department of Biochemical Engineering, School of Chemical Engineering & Technology, Tianjin University, Tianjin 300350, China; 2 Key Laboratory of Systems Bioengineering (Ministry of Education), Tianjin University, Tianjin 300072, China; 3 Department of Gastroenterology, Center of Tumor Immunology and Cytotherapy, Medical Research Center of The Affiliated Hospital of Qingdao University, Qingdao 266003, China; 4 Department of Chemical and Biological Engineering, University of Ottawa, Ottawa K1N 6N5, Canada

**Keywords:** Pancreatic cancer, nanobody therapeutic, intracellular antibody, HIF-1α, inhibitor, gemcitabine, chemosensitizer

## Abstract

**Objective:**

We aimed to develop a novel anti-HIF-1α intrabody to decrease gemcitabine resistance in pancreatic cancer patients.

**Methods:**

Surface plasmon resonance and glutathione S-transferase pull-down assays were conducted to identify the binding affinity and specificity of anti-HIF-1α VHH212 [a single-domain antibody (nanobody)]. Molecular dynamics simulation was used to determine the protein-protein interactions between hypoxia-inducible factor-1α (HIF-1α) and VHH212. The real-time polymerase chain reaction (PCR) and Western blot analyses were performed to identify the expressions of HIF-1α and VEGF-A in pancreatic ductal adenocarcinoma cell lines. The efficiency of the VHH212 nanobody in inhibiting the HIF-1 signaling pathway was measured using a dual-luciferase reporter assay. Finally, a PANC-1 xenograft model was developed to evaluate the anti-tumor efficiency of combined treatment. Immunohistochemistry analysis was conducted to detect the expressions of HIF-1α and VEGF-A in tumor tissues.

**Results:**

VHH212 was stably expressed in tumor cells with low cytotoxicity, high affinity, specific subcellular localization, and neutralization of HIF-1α in the cytoplasm or nucleus. The binding affinity between VHH212 and the HIF-1α PAS-B domain was 42.7 nM. Intrabody competitive inhibition of the HIF-1α heterodimer with an aryl hydrocarbon receptor nuclear translocator was used to inhibit the HIF-1/VEGF pathway *in vitro*. Compared with single agent gemcitabine, co-treatment with gemcitabine and a VHH212-encoding adenovirus significantly suppressed tumor growth in the xenograft model with 80.44% tumor inhibition.

**Conclusions:**

We developed an anti-HIF-1α nanobody and showed the function of VHH212 in a preclinical murine model of PANC-1 pancreatic cancer. The combination of VHH212 and gemcitabine significantly inhibited tumor development. These results suggested that combined use of anti-HIF-1α nanobodies with first-line treatment may in the future be an effective treatment for pancreatic cancer.

## Introduction

Although pancreatic cancer only contributes to 3% of all cancers, it is the fourth leading cause of cancer-related death in developed countries, with an overall 5-year survival of approximately 10%^1,2^. There is currently no specific, cost-effective biomarker that can easily and reliably diagnose early-stage pancreatic cancer, and most patients are asymptomatic at the time of early diagnosis until the disease develops to an advanced stage^3^. Although survival may increase to 35% after surgery, only 10%–15% of patients are eligible for surgery at diagnosis^4,5^. Both modified FOLFIRINOX and nab-paclitaxel plus gemcitabine, and capecitabine, and cisplatin regimens extend survival in pancreatic cancer patients^6,7^. However, chemotherapy has not resulted in satisfactory efficacy for pancreatic cancer because of poor responsiveness, side effects, and drug resistance caused by multiple factors^8,9^. Drug resistance to gemcitabine chemotherapy in pancreatic cancer patients is therefore an urgent problem. The stroma of pancreatic ductal adenocarcinoma creates a barrier for chemotherapy and generates a hypoxic microenvironment^10^. Thus, more effective medical treatments and biotherapeutic drugs are needed to treat patients with pancreatic adenocarcinoma.

HIF-1 plays crucial roles in regulating various downstream signaling pathways for tumor cells under hypoxic stress tolerance^11,12^. In the last decade, our group reported that several downstream factors regulated by HIF-1α were closely associated with tumor proliferation, angiogenesis, and the epithelial-mesenchymal transition^13–17^. Clinical trials and preclinical studies showed that targeting HIF-1α was an efficient strategy for healing pancreatic cancer^11,18–20^. Moreover, additional studies reported that high HIF-1α expression reduced sensitivity to gemcitabine, which is used to treat pancreatic adenocarcinoma and other cancers^9,21^.

Based on these findings, various HIF-1α inhibitors have been developed, with most of these being chemical inhibitors, siRNAs, and peptides^22,23^. However, side effects and microenvironment modulation effects in clinical trials have restricted their use^11,24^. Blocking the protein-protein interactions (PPIs) between HIF-1α and aryl hydrocarbon receptor nuclear translocator (ARNT) based on binding to the Per-ARNT-Sim (PAS) B domain of HIF-1α is therefore considered an effective strategy. However, HIF-1α is an intracellular target and full-length antibody for which certain immunological applications are mostly limited^25^. Single-domain antibodies (nanobodies, VHHs) are the smallest naturally occurring antibody fragments that can specifically bind to antigen epitopes. Furthermore, VHHs are stable at high temperatures, maintain their biological functions in tumor cells, and can be used for various immunological applications^25–27^. Because of their high efficiencies, great tumor penetrabilities, and excellent safety profiles, an adenovirus has been used as an engineering platform for gene therapy applications to intracellularly express VHH.

**Figure 1:**
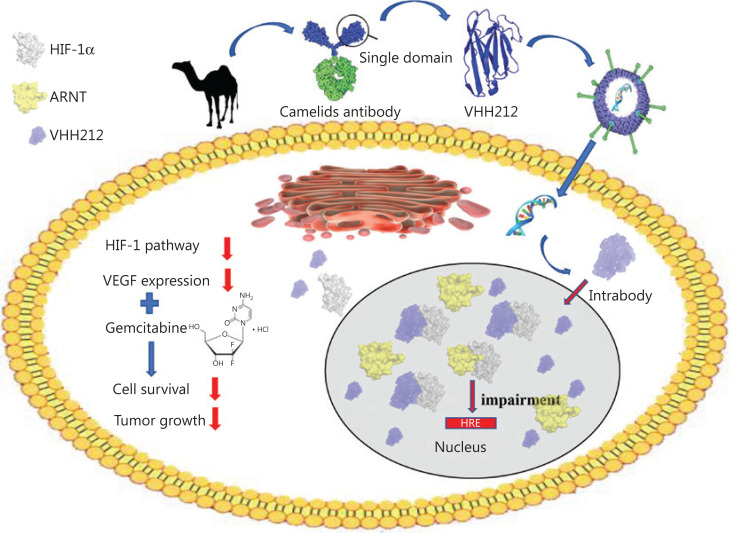
Graphical abstract of the study. A model summarizing how intrabody VHH212 sensitized the anti-tumor efficacy of gemcitabine by intracellularly targeting HIF-1α.

In the present study, we combined the VHH212-encoding adenovirus and gemcitabine for pancreatic cancer treatment and determined whether VHH212 enhanced the efficiency of gemcitabine by blocking HIF-1-mediated signaling (**Figure 1**). The results showed that the VHH212-encoding adenovirus may in the future be an effective treatment for pancreatic ductal adenocarcinoma (PDAC) patients.

## Materials and methods

### Cell culture and hypoxic treatment

The human PDAC cell lines, PANC-1 (RRID: CVCL_0480), BxPC-3 (RRID: CVCL_0186), were obtained from the Cell Culture Center, Peking Union Medical College (Beijing, China). MIA PaCa-2 (RRID: CVCL_0428) was obtained from the American Type Culture Collection (Manassas, VA, USA). All human cell lines were authenticated using short-tandem repeat (STR) profiling within the last 3 years. Mycoplasma contamination was excluded in these cell lines. PANC-1 and MIA PaCa-2 cell lines were cultured in Dulbecco’s Modified Eagle’s Medium (DMEM) (Gibco, Gaithersburg, MD, USA) supplemented with 10% fetal bovine serum (CellMax, Beijing, China) and the L-glutamine. BxPC-3 cell lines were cultured in RPMI 1640 medium (Gibco) supplemented with 10% fetal bovine serum (CellMax). Cells were grown at 37 °C in a humidified atmosphere of 95% air and 5% CO_2_. For hypoxic treatment, the cells were cultured in a modulator-incubator (Thermo Electron, Forma, MA, USA) in an atmosphere of 94% N_2_, 5% CO_2_, and 1% O_2_. The hypoxia conditions were used to mimic the average oxygen tension in pancreatic tumors.

### Reagents

Mouse monoclonal anti-HIF-1 alpha (ab1) and rabbit monoclonal anti-VEGF-A (ab52917) were obtained from Abcam (Cambridge, MA, USA). Mouse monoclonal β-actin antibody (60008-1-Ig), horseradish peroxidase (HRP)-conjugated Affinipure goat anti-mouse IgG (H + L) (SA00001-1), and HRP-conjugated Affinipure goat anti-rabbit IgG (H + L) (SA00001-2) were obtained from the Proteintech Group (Wuhan, China). Goat anti-mouse IgG (H + L) Cross-Adsorbed Secondary Antibody, and Alexa Fluor 594 (A-11005) were obtained from Invitrogen (Carlsbad, CA, USA).

### Plasmid construction and protein expression

**Figure 2:**
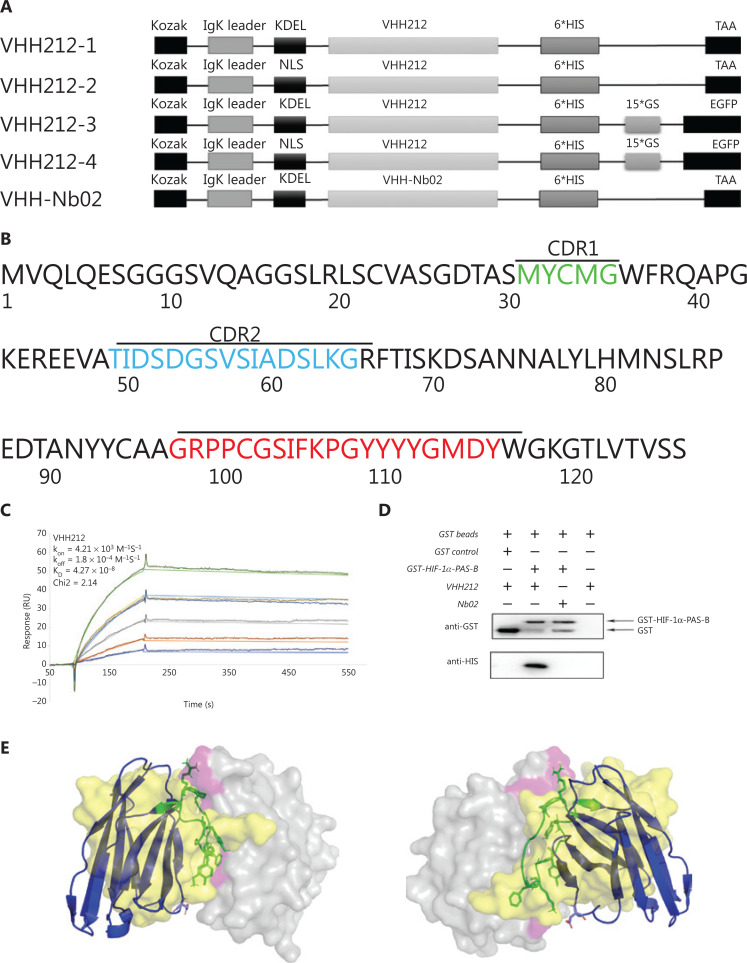
Engineered nanobody VHH212 has high binding affinity and specifically binds to the HIF-1α PAS-B domain. (A) Schematic representation of the sequence of the intrabody, from N-terminus to C-terminus. (B) Amino acid sequence of VHH212. The framework and complementarity-determining region sequences are defined according to the Kabat numbering scheme using the AbNum program. (C) A surface plasmon resonance sensorgram showing the interactions between the VHH212 and the HIF-1α PAS-B domain. The color lines represent the global fits of the raw data to a 1:1 bimolecular model. (D) Glutathione (GST) and GST-HIF-1α PAS-B fusion proteins were incubated with VHH212 or negative control Nb02, captured on glutathione-Sepharose beads, and analyzed. (E) Three-dimensional structure model of the HIF-1α PAS-B-VHH212 (K_D_ = 42.7 nM)/HIF-1α PAS-B-ARNT-PAS-B (K_D_ = 125 nM) interactions. The antigen (HIF-1α PAS-B domain) is shown as the gray translucent molecular surface, with the residues associated with VHH212 binding sites labeled in pink. The VHH212 is in a blue cartoon, with hotspots shown as a green stick model. The ARNT is shown as a yellow translucent molecular surface.

The human cDNA sequence of the HIF-1α-PAS-B domain was synthesized and subcloned into the pGEX-4T-1 prokaryotic expression vector (Amersham, San Ramon, CA, USA). The recombinant protein was produced in Rosetta (DE3) (Novagen, Darmstadt, Germany) and purified by affinity chromatography using a GSTrapFF column and ÄKTA primer plus (GE Healthcare, Chicago, IL, USA). The amino acid sequence of anti-HIF-1α-PAS-B-VHH212 (**Figure 2B**) (GenBank accession number: MK978327) was previously selected from a novel naive nanobody library by ribosome display technology and kept in our laboratory^28^. The VHH212 sequence was codon-optimized, synthesized, and subcloned into a pET-32a^+^ expression plasmid, with RBS/TATA box at the N-terminal and 6*His tag at the C-terminal, and was subsequently expressed in Origami™ B (DE3) (Novagen). Purification was performed by combining a HisTrapFF column and analytical size exclusion chromatographic Superdex™ 75 5/150 with ÄKTA explorer 100 (GE Healthcare).

**Figure 2A** shows that the sequence of intrabody VHH212 was codon-optimized, synthesized, and subcloned into pEGFP-N1, with a Kozak, IgK secretion leader, nuclear localization sequence (NLS) or Lys-Asp-Glu-Leu sequence (KDEL) leader peptide at the N-terminal and 6*His tag at the C-terminal by GENEWIZ (Beijing, China). An anti-CD47 nanobody, Nb02 (GenBank accession number: MK780744), was subcloned into pEGFP-N1 as a negative control. Subsequently, the amino acid sequence of VHH212-1 was amplified using the polymerase chain reaction (PCR) and cloned into a pAD/CMV/V5-DEST plasmid using a Gateway cloning reaction (Thermo Fisher Scientific, Waltham, MA, USA). The recombinant intrabody-encoding adenovirus was essentially produced according to the manufacturer’s instructions, while high titer virus stocks were produced and purified by R&S Biotechnology (Shanghai, China). The pGL3-6*HRE was constructed, synthesized, and 6* TCGAGGCCCTACGTGCTGTCTCACACA GCCTGTCTGACG was subcloned into a pGL3-basic plasmid by GENWIZ.

### Binding affinity measurements using surface plasmon resonance (SPR)

SPR measurements were performed using a Biacore 3000 instrument (GE Healthcare) at 25 °C. A purified HIF PAS-B domain protein (2 μg/mL in 10 mM HEPES, pH 7.4, 150 mM NaCl, 3 mM EDTA, 0.005% P20) was covalently bound to a CM5 sensor chip at an immobilization density of approximately 350 resonance units (RUs). In addition, an unrelated biotinylated protein was immobilized with an RU value matching that of the reference surface to control for nonspecific binding. IMAC-SEC-purified VHH212 was serially diluted using BIAcore running buffer [10 mM HEPES, pH 7.4, 150 mM NaCl, 3 mM EDTA, 0.05% (v/v) P20] while taking measurements at a flow rate of 40 μL/min. A running buffer without VHH was then passed over the chip to allow spontaneous dissociation at the same flow rate. After each run, the sensor chip was regenerated by injecting 10 mM glycine, pH 2.0. Affinities were calculated using the Biacore 3000 evaluation software (GE Healthcare) with a 1:1 Langmuir binding model.

### Homology modeling, molecular docking, and hotspot analysis

MODELLER, version 9.19 (https://salilab.org/modeller/9.19/manual/), was used to build the structural model of VHH212. The amino acid sequence of VHH212 was searched in the protein databank with BLAST (http://blast.ncbi.nlm.nih.gov/Blast.cgi), with an E-value cutoff of 10.0 and sequencing identity cutoff of 90%, and a 50 ns molecular dynamics simulation of the model was performed to eliminate unreasonable structures.

AutoDock Vina (http://vina.scripps.edu/) was used for molecular docking, and the VHH212-HIF-1α-PAS-B domain complex structure was predicted using standard protocols. The atomic coordinates of the HIF-1α-PAS-B domain were obtained from the HIF-1/ARNT PAS-B complex crystalline structure (PDB ID: 4H6J)^29^. Hotspots of VHH212 within a 5 Å distance of the HIF-1α-PAS-B domain were analyzed using PyMOL and PIC-SERVER (http://pic.mbu.iisc.ernet.in).

### Thermal stability measurements

To determine the thermal stability of the intrabody, real-time fluorescence quantitative PCR (qPCR) was conducted to measure the thermal denaturation and melting curves of VHH212. The qPCR was conducted using the Roche LightCycler® 480 II real-time PCR instrument (Roche, Basel, Switzerland). Samples were prepared in a total volume of 20 μL containing 2.0 μM VHH212 in a sample buffer [50 mM HEPES, pH 7.5, 300 mM NaCl, 0.5 mM TCEP, and 5× SYPRO Orange (Sigma-Aldrich, St. Louis, MO, USA)] and were monitored in 96-well PCR plates, with excitation at 465 nm and emission of 580 nm. Thermal denaturation temperature was analyzed using LC480 software 1.5.0 with a LightCycler Thermal Shift Analysis plug-in (Roche). Each sample was measured in quintuplicate and the average arithmetic value was calculated.

### Transient transfection experiments and reporter assays

All the transient transfections were performed as per the manufacturer’s instructions using Lipofectamine™ 2000 (Invitrogen). For the Luciferase Reporter Assay, MIA PaCa-2 and PANC-1 cells were plated on 24-well plates and cultured until they were almost 60% confluent. The pGL3-Basic (Promega, Madison, WI, USA) and pGL3-6*HRE were chosen as negative and positive controls, respectively. Reporter plasmids (500 ng) were co-transfected with an internal control pRL-SV40 (10 ng) into PANC-1 or MIA PaCa-2 cells. The cells were then transfected with 500 ng of pEGFP-N1 and pcDNA-3.1, pEGFP-VHH212-2 and pcDNA-HIF-1α-OE, or pEGFP-N1 and pcDNA-HIF-1α-OE plasmids per well^14^. Cells were then harvested 48 h after transfection, and the two luciferase activities were measured consecutively using the dual-luciferase reporter Assay System (Promega). All data were normalized to Renilla luciferase expression using at least 3 independent experiments.

### RNA extraction, cDNA synthesis, and real-time PCR detection

Total RNA was isolated from pancreatic cancer cells using the TRIzol™ reagent (Invitrogen), and reverse transcription was performed using the RevertAid First Strand cDNA Synthesis Kit (Thermo Fisher Scientific), as per the manufacturer’s instructions. For quantitative comparisons, cDNA samples were analyzed by real-time PCR using the TB Green® Premix Ex Taq™ (Takara, Shiga, Japan) on the CFX96 Real-Time PCR System (Bio-Rad, Hercules, CA, USA). PCR cycling conditions were 95 °C for 30 s, 40 cycles of 95 °C for 5 s, 60 °C for 30 s, and then use of the final melting curve program. All experiments were conducted in triplicate and the mean values were used for quantitation. Relative expression values were calculated using the equation R equal to 2^– (ΔCt target–ΔCt reference)^, where the ΔCt target was the fractional threshold cycle of the target gene in the experimental group minus that in the control group, and the Ct reference was the fractional threshold cycle of β-actin.

Primers for VEGF-A were forward: 5′-AAG GAG GAG GGC AGA ATC AT-3′ and reverse: 5′-CAC ACA GGA TGG CTT GAA GA-3′; primers for HIF-1α were forward: 5′-GTG TTA TCT GTC GCT TTG AGT C-3′ and reverse: 5′-GTC TGG CTG CTG TAA TAA TGT TC-3′; primers for β-actin were forward: 5′-TCA TCA CCA TTG GCA CTG AG-3′ and reverse: 5′-CAC TGT GTT GGC GTA CAG GT-3′.

### Western blot analyses and GST pull-down assays

The treated cells were lysed using a SDS lysis buffer supplemented with a protease inhibitor cocktail (Sigma-Aldrich). The protein concentration was quantified using a BCA Protein Assay Kit (Solarbio, Beijing, China) and equal amounts of total protein were detected using a 12% SDS-polyacrylamide gel. The proteins were then transferred to a polyvinylidene difluoride blotting membrane (GE Healthcare) and probed with target antibodies at 4 °C overnight. The antigen-antibody complex was detected by incubating the membranes with HRP-conjugated secondary antibody at room temperature for 2 h; the complex was visualized using an ECL Western blot substrate (Solarbio).

For the GST pull-down assay, purified GST-HIF-1α-PAS-B or GST was incubated with Glutathione Sepharose 4B beads (GE Healthcare) at 4 °C overnight. The beads were washed 3 times with reaction buffer, and incubated with purified VHH212-6*His at 4 °C overnight^30^. The beads were then washed 3 times and boiled for 5 min at 95 °C. The supernatants were subjected to 10% SDS-PAGE and Western blot.

### Immunofluorescence confocal microscopy

Immunofluorescence staining of HIF-1α was conducted to evaluate the efficiency of the subcellular location capacity of the intrabody. MIA PaCa-2 cells were transfected with either pEGFP-N1, pEGFP-VHH212-3, or pEGFP-VHH212-4. After incubation for 48 h at 37 °C under hypoxic conditions, the cell on slides were washed 3 times with phosphate-buffered saline (PBS) and fixed for 10 min in 4% paraformaldehyde at room temperature. The fixed cells were permeabilized with 0.2% Triton X-100 for 10 min and blocked with 2% goat serum for 1 h at room temperature. The slides were then incubated with the primary antibody, secondary antibody, and 4′,6-diamidino-2-phenylindole as previously described^31^. The images were captured using a TCS SPE confocal system (Leica Microsystems, Mannheim, Germany).

### Cell proliferation assay

To assess the potential cytotoxic effects of intrabodies, the cell viability of PANC-1, BxPC-3, and MIA PaCa-2 cells were measured. The pEGFP-N1, pEGFP-NSL-VHH212, pEGFP-KDEL-VHH212, and pEGFP-VHH2 were transfected into cancer cell lines using Lipofectamine 2000. After incubation for 48 h at 37 °C under hypoxic conditions, 10 μL CCK-8 (Solarbio) was added to a 96-well plate. After an additional 2 h of incubation at 37 °C with CCK-8, the absorbance was measured at 450 nm using a Synergy HT (Bio Tek, Winooski, VT, USA).

### Wound migration assay

Transfected or wild-type PANC-1 and MIA PaCa-2 cells were plated on 6-well plates until 90% confluent, and cell monolayers were scraped using a pipette tip. PBS was then used to remove cell debris. The monolayers were treated with gemcitabine (100 nM) and/or digoxin (40 nM) for 48 h, and the migrating cells were observed using an Axio A1 microscope (Carl Zeiss, Jena Germany). The wound healing area was then measured using ImageJ software (National Institutes of Health, Bethesda, MD, USA).

### Transwell invasion assay

Cellular invasion was measured using Transwell permeable support systems (Corning, NY, USA). The inserts were coated with 11.1% Matrigel (BD Biosciences, San Jose, CA, USA) and incubated overnight at 37 °C before cell seeding. The treated PANC-1 and MIA PaCa-2 cells were seeded into the upper Transwell chambers in serum-free DMEM containing gemcitabine (100 nM) and/or digoxin (40 nM) for 72 h. The lower chambers contained 10% fetal bovine serum (FBS) without drugs. The cells that invaded the 8.0 μm porous polyethylene tetraphthalate membrane were identified by staining with 0.5% Crystal Violet and counted using ImageJ software.

### Colony formation assay

Approximately 500 transfected or wild type cells were plated on 6-well plates and incubated overnight at 37 °C. The cells were then treated with gemcitabine (100 nM), digoxin (40 nM), or in a combination of both for 24 h. After changing with drug-free and FBS-free medium, the cells were further incubated for 2 weeks at 37 °C, then fixed with 4% formaldehyde and stained with 0.5% Crystal Violet.

### Xenograft models

BALB/c nude mice (8–10 weeks of age; 20–22 g) were purchased from Vital River Laboratory Animal Technology (Beijing, China). The subcutaneous xenograft model of pancreatic cancer was established by subcutaneously injecting 10^7^ PANC-1 cells in a 0.2 mL PBS solution containing 10% FBS into the right flank of nude mice. The BALB/c nude mice bearing the PANC-1 tumor model were acclimated at 25 °C and 55% of humidity under natural light/dark conditions, with standard mice chow and water available *ad libitum*. At 14 days after inoculation, tumors grew to an average of 100 mm^3^. At this time, 30 mice were randomly distributed into 5 groups. As shown in **Figure 6A**, in the adenovirus treatment groups, 8 × 10^8^ IU intrabody-encoding adenoviruses (pAd-VHH212-2) or control adenoviruses (pAd-CMV-V5-DEST) were injected intratumorally once a week. In the gemcitabine treatment groups, an intraperitoneal injection of 50 mg/kg gemcitabine (Gemzar; Eli Lilly, Indianapolis, IN, USA) was administered twice a week. The same doses of gemcitabine and adenoviruses were administrated in the combined treatment group, and an intraperitoneal injection of normal saline was used as the negative control. The tumor volume was measured over the skin and calculated as previously described^32^. All animals were sacrificed 1 week after the last drug administration, followed by weighing of the tumors.

The mice were bred at an animal care facility certified by the Tianjin Management Committee of Laboratory Animals in the Institute of Radiation Medicine Chinese Academy of Medical Sciences. All animal experiments (Approval No. IRM-DWLL-2019-126) were approved by the Animal Ethics Committee of the Chinese Academy of Medical Science and Peking Union Medical College.

### Immunohistochemical and hematoxylin & eosin (H&E) staining

To evaluate HIF-1α/VEGF *in vivo* downregulation, tumor tissues were fixed in 10% formaldehyde and embedded in paraffin. The paraffin sections (4 mm) were dewaxed in xylene and rehydrated using a graded series of ethanol. The tumor tissue sections were then subjected to routine H&E and immunohistochemical staining for HIF-1α and VEGF-A. The resulting images were captured using an Axio Observer 7 microscope (Zeiss).

### Statistical analysis

All data are expressed as the mean ± standard error of the mean (SEM) using Prism 7.0 software (GraphPad, San Diego, CA, USA). All data were derived from at least 3 independent experiments. Statistical analyses were conducted using Student’s *t-*test or analysis of variance using GraphPad Prism 7.0 software. A value of *P* < 0.05 was considered statistically significant.

## Results

### VHH212 has high binding affinity to the PAS-B domain

A nanobody is the smallest antigen-binding fragment with a complete function, and is a type of neutralizing antibody that does not cause antibody-dependent cell-mediated cytotoxicity. A high binding affinity is therefore essential for a nanobody to compete with the natural subunit or receptor. In the present study, the affinity constant of VHH212 was determined using SPR measurements. The binding affinity of VHH212 and the HIF-1α PAS-B domain was 42.7 nM, with a chi square (χ^2^) equal to 2.14 (**Figure 2C**).

### Direct interaction of VHH212 with HIF-1α

Direct interaction of the HIF-1α PAS-B domain and VHH212 *in vitro* was determined using a GST pull-down assay. The results indicated that anti-HIF-1α VHH212 was pulled down by the HIF-1α PAS-B domain, whereas the anti-CD47 Nb02 was not (**Figure 2D**).

The interaction between HIF-1α and VHH212 was confirmed by *in silico* simulation. A three-dimensional antigen-antibody complex was constructed based on homology modeling and molecular docking. The mechanism of competitive inhibition by anti-HIF-1α VHH212 and the interaction between HIF-1α and ARNT were revealed by molecular dynamic simulations. As shown in **Figure 2E**, VHH212 occupied the PAS-B domain of HIF-1α, which originally interacted with ARNT. **Table 1** shows that the active residues of the HIF-1α and VHH212 complex were identified. Protein-protein ionic interactions and main chain-main chain hydrogen bonds formed a tight junction protein interaction between HIF-1α and VHH212, which was beneficial for the stabilization of the protein complex structure. In this case, anti-HIF-1α VHH212 competitively inhibited the HIF-1 pathway by neutralizing HIF-1α and blocking the HIF-1 subunit protein-protein interaction.

**Table 1:** Binding site comparisons between the HIF-1α PAS-B/VHH212 complex and HIF-1α PAS-B/ARNT PAS-B

PPI	HIF-1α and VHH212 complex				4H6J^29^			
	VHH212		HIF-1α		ARNT		HIF-1α	
	Position	Residue	Position	Residue	Position	Residue	Position	Residue
PPII	61	ASP	256	ASP	362	ARG	245	GLU
			257	GLU	379	ARG		
			258	ARG	366	ARG	256	ASP
PPMMHB	109	GLY	337	CYS				
	110	TYR	244	SER				
PPAAI	110	TYR	254	TYR	375	PHE	254	TYR
PPMSHB	99	ARG	326	ASN	440	ARG	325	TYR
			327	THR				
			328	LYS				
PPSS	61	ASP	258	ARG	245	GLU	362	ARG
HB	110	TYR	256	ASP			379	ARG
PPHI	100	PRO	324	ILE	243	LEU	364	ILE
			325	TYR			375	PHE
	106	PHE	338	VAL			458	ILE
			340	TYR	254	TYR	364	ILE
			342	VAL			375	PHE

PPI, protein-protein interactions; PPII, protein-protein ionic interactions; PPMMHB, protein-protein main chain-main, chain hydrogen bonds; PPAAI, protein-protein, aromatic-aromatic interactions; PPMSHB, protein-protein, main chain-SIDE chain hydrogen bonds; PPSSHB, protein-protein side chain-side, chain hydrogen bonds; PPHI, protein-protein hydrophobic interactions.

### The high thermal stability of VHH212

**Figure 3:**
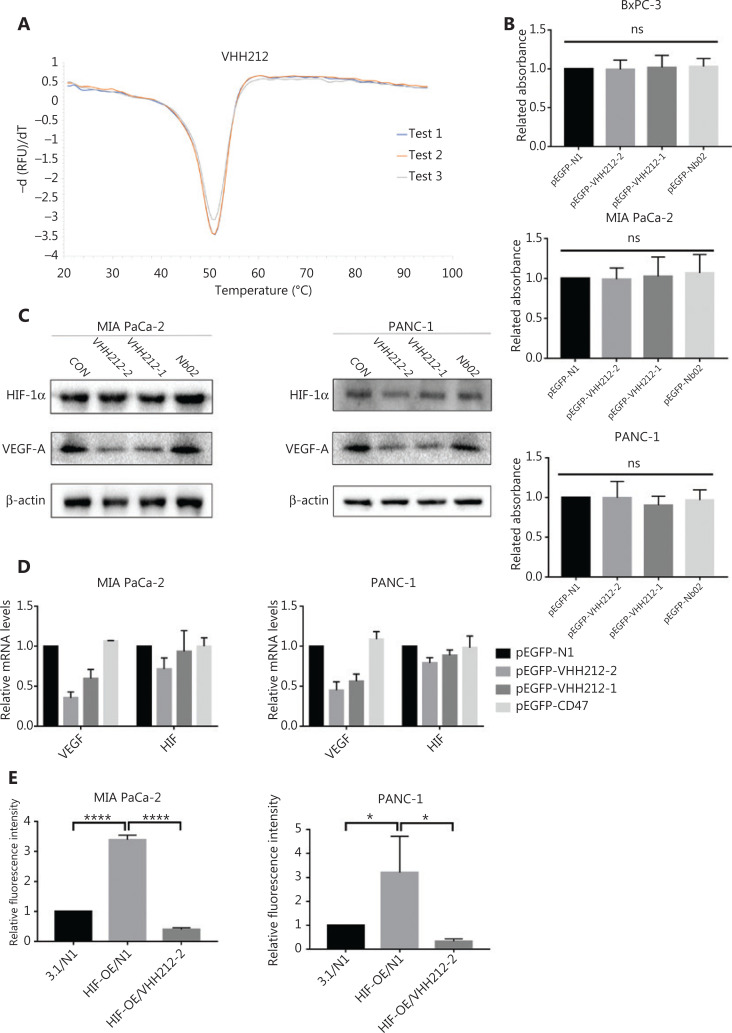
VHH212 shows excellent thermal stability, low cytotoxicity, and the capacity to competitive inhibit the HIF-1 pathway. (A) An experiment to measure the thermal stability of VHH212. The Tm values were determined by melting curves using qPCR, and the value was average from 3 independent experiments. (B) Intrabodies do not have direct cytotoxic properties *in vitro*. The CCK8 assay was used to determine cell viability, and did not show a statistical difference between intrabodies and the vector control. (C) Western blot analysis of pancreatic cancer cell lines after hypoxic treatment. MIA PaCa-2 and PANC-1 cells were transfected with intrabodies and incubated under hypoxia (36 h), and total protein extracts were processed for Western blot using anti-HIF-1α, anti-VEGF-A, and anti-β-actin antibodies. (D) Quantitative real-time RT-PCR of pancreatic cancer cell lines after hypoxic treatment. MIA PaCa-2 cells were transfected, treated with hypoxia, and lysed in TRIzol for RNA extraction and analyzed for the mRNA expression of HIF-1α and VEGF by quantitative real-time RT-PCR. Data are expressed as the mean ± SEM. Columns, the mean of three experimental determinations; bars, standard deviation. (E) Luciferase analysis of MIA PaCa-2 and PANC-1 cells. The cells were transfected as described above. Relative luciferase analysis used the Dual-Luciferase Reporter Assay System, and the Renila vector was transfected as an internal control. Results are expressed as fold induction relative to cells transfected with the control vector (pcDNA3.1) after normalization to Renila activity. Columns, mean of 3 independent experiments; bars, standard deviation. (*P < 0.05) *vs.* the control. 3.1, pcDNA-3.1; N1, pEGFP-N1; VHH212, pEGFP-VHH212-2; HIF-OE, pcDNA-HIF-1α-OE. ****P < 0.0001.

To determine the thermodynamic stability of VHH212, SYPRO orange dye was used in the ThermoFlour assay using a LightCycler® 480 II (Roche). SYPRO orange has a high fluorescence intensity when reacting with hydrophobic regions of proteins^33^. With increasing temperature, the fluorochrome bound to the exposed hydrophobic regions of the unfolding protein, and the fluorescence intensity simultaneously increased. In contrast, the fluorescence intensity decreased after denaturation. As shown in **Figure 3A**, the Tm value of the VHH212 was 50.75 °C.

### VHH212 is not cytotoxic to PDAC cell lines

A CCK-8 cell proliferation assay was used to measure cell viabilities after transient transfections with pEGFP-N1, pEGFP-VHH212-1, pEGFP-VHH212-2, and pEGFP-Nb02. The results showed that the intrabody did not have direct cytotoxic effects on tumor cells. MIA PaCa-2 and PANC-1 cells were transfected with plasmids and cultured under hypoxic conditions, followed by determination of their viabilities. **Figure 3B** shows that no significant difference in the viability of tumor cells was observed, irrespective of exposure to intrabody VHH212, control nanobody (Nb02), or the negative control. The results suggested that the therapeutic effect was not based on direct cytotoxicity to tumor cells.

### VHH212 inhibits the HIF-1 pathway

As shown in **Figure 3C**, the proteins extracted from MIA PaCa-2 and PANC-1 cells transfected with pEGFP-N1, pEGFP-VHH212-1, pEGFP-VHH212-2, or pEGFP-Nb02 were analyzed using Western blot. The cells were harvested and intrabodies were detected with an anti-His-tag antibody. Intrabody VHH212 bound to the PAS-B domain of HIF-1α to competitively inhibit HIF-1α- ARNT heterodimer formation to inhibit the mRNA and protein expressions of VEGF-A in MIA PaCa-2 and PANC-1 cells (**Figure 3C–3D**). To further confirm that intrabody binding to the HIF-1α PAS-B domain inhibited the HIF-1 pathway, luciferase analysis was conducted. The hypoxia-response element (HRE) promoter activity (pGL3-6*HRE) increased ∼3-fold in the presence of the HIF-1α overexpression plasmid pcDNA-HIF-1α-OE, when compared with the pEGFP-N1 and pcDNA-3.1 groups Furthermore, the promoter activity decreased ∼8.6-fold when pEGFP-VHH212-2 and pcDNA-HIF-1α-OE were co-transfected. The results showed that VHH212 significantly inhibited the formation of the functionally active HIF-1 transcription factor complex (**Figure 3E**).

### Intrabody expression and subcellular location in tumor cells

**Figure 4:**
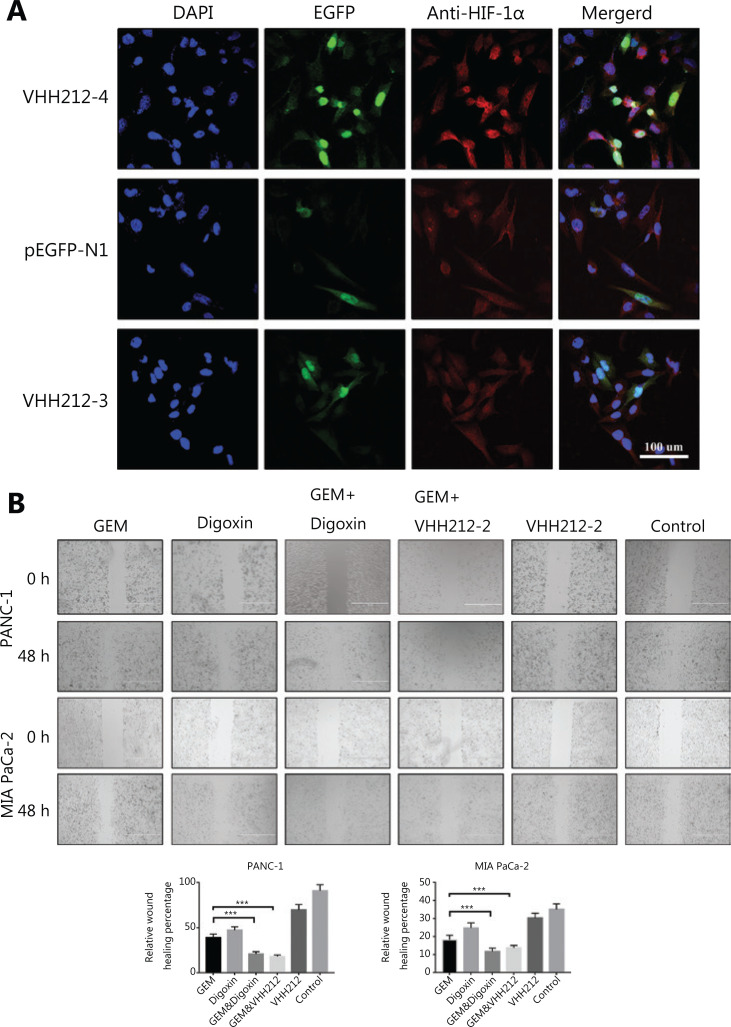
VHH212 inhibits cell migration and specific subcellular localization. (A) Confocal images of MIA PaCa-2 cells transfected with pEGFP-N1, pEGFP-VHH212-1, and pEGFP-VHH212-2, then stained with 4′,6-diamidino-2-phenylindole (blue), EGFP (green), and HIF-1α (red) with a magnification of 40×. (B) Inhibition of pancreatic cancer cell migration. Transfected or wild type PANC-1 and MIA PaCa-2 cells were plated in 6-well plates until 90% confluent. The cell layer was scratched and treated with gemcitabine (100 nM) and/or digoxin (40 nM) for 48 h. ***P < 0.001.

Immunofluorescence testing indicated that the intrabody with the EGFP tag was well-expressed in tumor cells and allowed visualization in living cells. The intrabody with an NLS tag bound to endogenous stabilized HIF-1α, and a strong nuclear EGFP fluorescence was detected (**Figure 4A**). However, this phenomenon was not observed in tumor cells transfected with VHH212-3 and the null vector.

### VHH212 inhibits proliferation, invasion, and migration of pancreatic cancer cell lines

**Figure 5:**
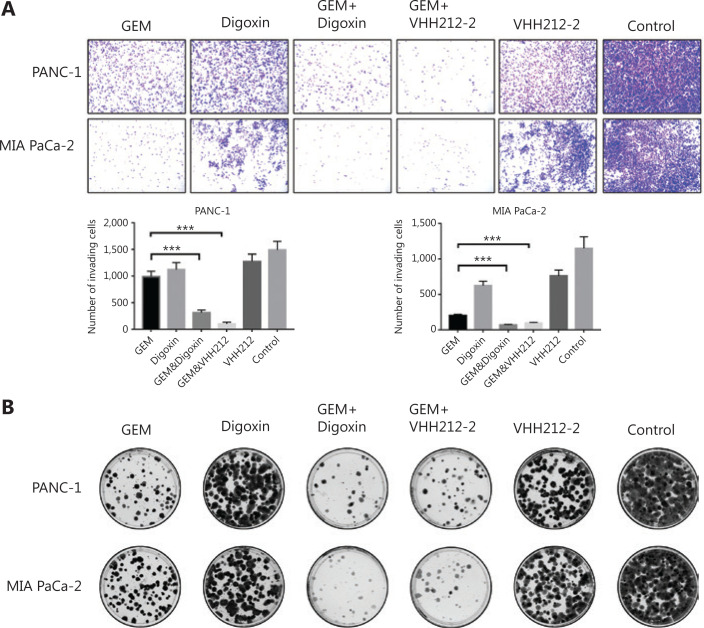
VHH212 suppressed the proliferation and metastasis of tumor cells. (A) Inhibition of pancreatic cancer cell invasion. Treated PANC-1 and MIA PaCa-2 cells were seeded into upper Transwell chambers in serum-free Dulbecco’s Modified Eagle’s medium containing gemcitabine (100 nM) and/or digoxin (40 nM) for 72 h. The lower chambers contained 10% fetal bovine serum medium without drugs. After incubation for 72 h, the cells were stained with 0.5% Crystal Violet. (B) Colony formation assay. Five-hundred treated PANC-1 and MIA PaCa-2 cells were plated in 6-well plates and incubated for 14 days. ***P < 0.001.

Because HIF-1α plays crucial roles in the proliferation, invasion, and migration of PDAC, especially in metastasis, cell migration assays were conducted using PANC-1 and MIA PaCa-2 cells. In both cell lines, migration was significantly inhibited by VHH212 and gemcitabine, and digoxin and gemcitabine combined treatment was better when compared with either agent alone, especially in PANC-1 cells (**Figure 4B**). Furthermore, the invasive abilities of these 2 cell lines were determined using a Transwell assay. Single agent gemcitabine caused 33% inhibition in PANC-1 cell lines, whereas VHH212 and gemcitabine, and digoxin and gemcitabine combined treatment resulted in 78% and 92% inhibition, respectively (**Figure 5A**). Furthermore, cell colony formation assays showed that both anti-HIF-1α VHH212 and digoxin combination with gemcitabine dramatically inhibited cell proliferation in pancreatic cancer cell lines (**Figure 5B**).

### VHH212 inhibits tumor proliferation in a xenograft pancreatic cancer model

**Figure 6:**
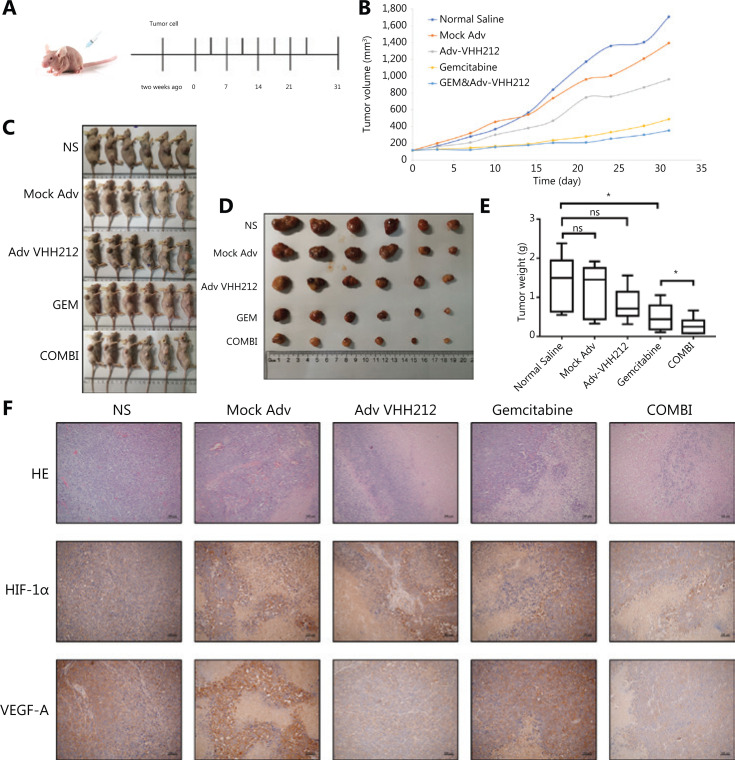
VHH212 sensitized gemcitabine anti-tumor effects *in vivo*. (A) The schematic illustration of the establishment of the PANC-1 xenograft model and administration strategy. (B) The tumor volume of mice treated with different treatment strategies. (C) Images of tumor-bearing mice on day 31. (D) An image of tumors isolated from tumor-bearing mice on day 31. (E) Average tumor weights. (F) H&E staining of tumor tissues and the expression of HIF-1α and VEGF-A were confirmed using an immunohistochemistry assay in tumor tissues. *P < 0.05.

The anti-tumor effects of anti-HIF-1α-VHH212 and gemcitabine co-treatment were then determined using a PANC-1 xenograft model. The tumor volumes were monitored twice a week using digital calipers (**Figure 6B–6C**). **Figure 6D–6E** shows that tumor growth inhibition was significantly higher with combined treatment, when compared with gemcitabine alone. Intrabody-encoding adenovirus treatment did not show sufficient antitumor activity in the PANC-1 xenograft model. These results indicated that VHH212 sensitized gemcitabine anti-tumor effects *in vivo*. Compared with tumor inhibition in the normal saline group, those in the Mock Adv, Adv-VHH212, gemcitabine, and the co-treatment groups were 12.56%, 41.58%, 64.89%, and 80.44%, respectively.

**Figure 6F** shows that co-treatment increased necrotic regions in tumors, as determined using H&E and immunohistochemical staining. The tumor tissue in the normal saline group was rich in glandular cavities, and inflammation reactions occurred in the mock adenovirus and intrabody-encoding adenovirus treatment groups. Large areas of necrosis were observed in the gemcitabine treatment group, whereas fibrous capsule formation was observed in the co-treatment group. Furthermore, intrabody-encoding adenovirus treatment decreased the expression of VEGF-A in tumors, as determined by immunohistochemical analysis.

## Discussion

Caplacizumab, the first nanobody-based medicine, has become a first-class orphan drug to be launched in both Europe and the U.S. for the treatment of acquired thrombotic thrombocytopenic purpura^34^. This resulted because of the economic and research results using Nanobodies®, which have revived interest in developing single-domain antibody-based biotherapeutics^35^. Furthermore, the unique advantages of nanobodies make them a potential candidate for the treatment of unexpected infectious diseases^36^.

Gemcitabine plus nanoparticle albumin-bound paclitaxel is used as a first-line treatment in advanced pancreatic cancer patients, but has a median overall survival of less than 10 months^37^. Most patients do not respond to gemcitabine treatment, and among those who do, almost all acquire drug resistance^38^. The tumor microenvironment of PDAC is characterized by desmoplasia, leading to a hypoxic environment and acting as a barrier for chemotherapy. Gemcitabine resistance caused by these factors is therefore a serious clinical issue.

A hypoxic microenvironment has been correlated with tumor cell metastasis, invasiveness, angiogenesis, and chemoresistance, all of which contribute to poor long-term overall survival^39,40^. HIF-1α, as a principal regulatory response to hypoxia tolerance, has been shown to mediate genes associated with pancreatic cancer progression, prognosis, and surgical outcomes^13,14,16,41,42^. HIF-1α overexpression is correlated with aberrant p53 accumulation, and promotes the development of pancreatic cancer by activating downstream signaling pathways^43,44^. Previous studies have shown that high HIF-1α expression levels decreased the sensitivity to gemcitabine, which has been used in pancreatic cancer treatment^21^. Gemcitabine treatment has also been associated with the accumulation of HIF-1α in the nucleus^45^. Thus, various strategies have been developed for the suppression of pancreatic cancer invasiveness and proliferation using suppression of HIF-1α^22,46^.

Numerous chemical compounds have been identified as functional inhibitors of the HIF-1 signal pathway, which decrease HIF-1α expression levels^47–49^. However, low specificity to the target protein can cause unacceptable cytotoxicity, side effects, drug resistance, and other negative clinical outcomes. RNA interference technology has also been used for the downregulation of HIF-1α, but its low efficacy has limited its application^50^. The CRISPR/Cas9 system is a powerful approach for inhibiting tumor metastasis, but off-target effects limit its further clinical use^11,51^. Furthermore, although antibodies have successfully been used for targeting cell surfaces or soluble antigens^52,53^, monoclonal antibodies are not effective in targeting intracellular antigens^54^.

Based on these considerations, the unique characteristics of nanobody-based biotherapeutics have made it possible to address these intractable issues. Previous studies have shown that the binding affinity constant of HIF-1α to ARNT is approximately 125 nM^29^. Furthermore, an anti-HIF-1α nanobody VHH212 has shown excellent thermal stability (**Figure 3A**), low cytotoxicity (**Figure 3B**), and high binding affinity (K_D_ = 42.7 nM) as a competitive inhibitor. Preclinical animal studies and clinical trials have shown that nanobody-based biotherapeutics are well tolerated in patients, with a superior therapeutic window^55,56^. Furthermore, VHH can be expressed in mammalian cells and maintains binding capacities that target cytoplasmic or nuclear antigens as intrabodies^26,57^. Good stability and tissue penetration abilities provide the opportunity to overcome the barrier of the tumor microenvironment in PDAC patients.

Based on these results, we hypothesized that blocking the HIF-1 pathway using VHH212 may enhance the efficacy of gemcitabine in pancreatic cancer patients. In the present study, VHH212 effectively inhibited VEGF-A expression *in vitro* and *in vivo*, and suppressed vessel growth and vascular permeability in solid tumors (**Figure 6F**). Simultaneously, there were two clinical trials in progress using digoxin as a chemical inhibitor of HIF-1α in pancreatic cancer. In the present study, we showed that VH212 combined with gemcitabine achieved lower cytotoxicity and the same inhibitory effect as digoxin co-treatment in pancreatic cancer cell lines.

For the first time, both these results showed that a novel nanobody-based biotherapeutic intracellular antibody, VHH212, directly bound to the HIF-1α PAS-B domain to inhibit the HIF-1 pathway and enhance the efficacy of gemcitabine chemotherapy *in vitro* and *in vivo*.

Adenovirus is a widely used vector for cancer gene therapy with a high capacity for transgene expression^58^, and adenovirus gene therapy drugs have shown great tolerance and lower toxicity in most clinical studies^59^. Using an adenoviral vector to express a nanobody would therefore be an ideal alternative due to its high gene expression, high titer, and mature production technology. The VHH212-encoding adenovirus designed in this study has been shown to have 3 levels of anti-tumor effects: (1) as a foreign body, the adenovirus vector activated the immune response of the tumor microenvironment; (2) intrabody VHH212 competitively inhibited HIF-1 pathways; and (3) its targeted HIF-1α enhanced the anti-tumor effects of gemcitabine.

However, several studies are required to improve the application of VHH212 in pancreatic cancer treatments. For example, with the development of data accumulation and algorithm optimization, high affinity nanobodies can be explored using virtual mutation screening technology^28,60^. In addition, synthetic biology is driving a new era of nanobody-based drug delivery systems for digestive system diseases^61^.

## Conclusions

We developed a novel anti-HIF-1α nanobody and demonstrated the function of VHH212-encoding adenovirus in a preclinical murine model of PANC-1 pancreatic cancer. The combination of VHH212 with gemcitabine significantly inhibited tumor development, suggesting that combined anti-HIF-1α nanobodies for first-line treatment could be a potent therapeutic regimen.
